# Supplementary material for: Active metabolism unmasks functional protein–protein interactions in real time in-cell NMR
Source: Commun Biol. 2020 May 21;3:249. doi: 10.1038/s42003-020-0976-3 (PMC7242440; doi:10.1038/s42003-020-0976-3)
Supplement: Supplementary file 1 — Supplementary Information [file 42003_2020_976_MOESM1_ESM.pdf]

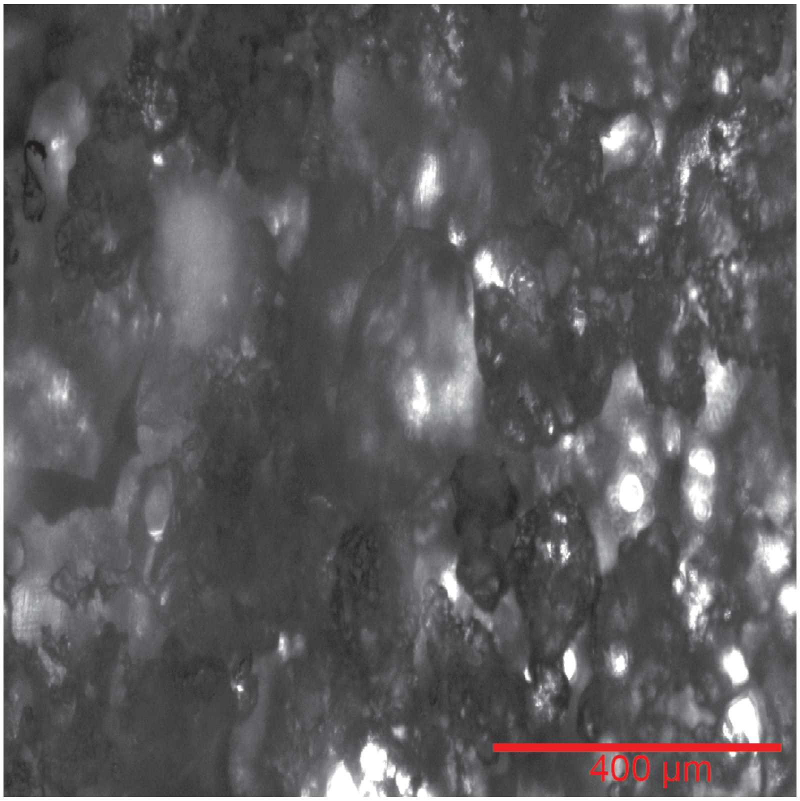

Supplementary Figure 1. Microscopic image of the hydrophilic microporous polyethylene diffuser.

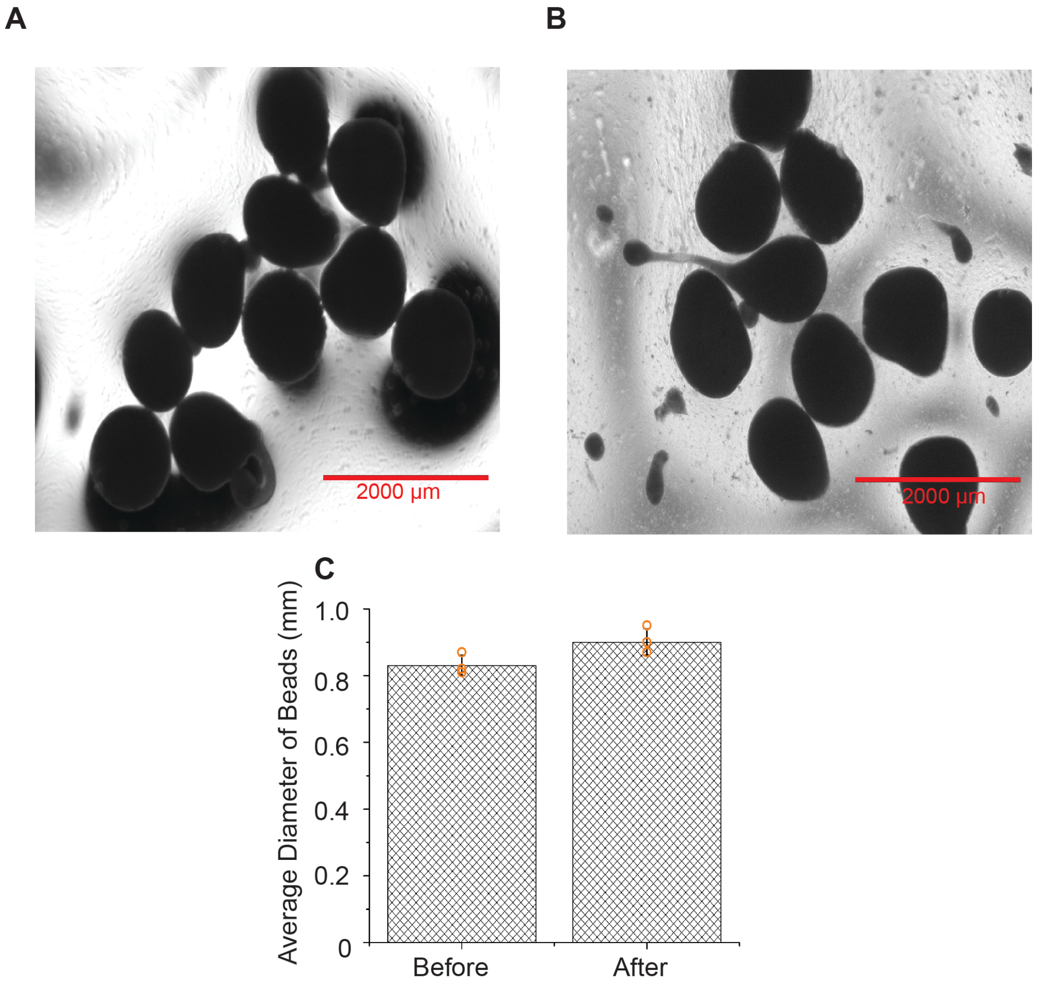

Supplementary Figure 2. Alginate beads with encapsulated cells before (A) and after (B) 24 hour long in-cell NMR experiment using bioreactor. C) Beads placed in the bioreactor had an average diameter of 0.83 ± 0.03 mm; after 24 hours, the diameter was increased to 0.90 ± 0.04 mm. The error bars give the standard error of the mean for each data point.

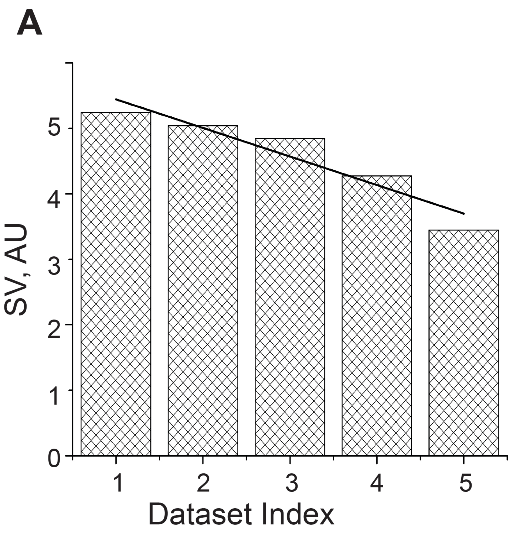

Supplementary Figure 3. Random noise results in SVs that are fit with a linear correlation greater than 0.9. To simulate experimental noise, a matrix of the same size as that produced by RT STINT NMR containing random signal intensity values between -1 and 1 was generated by using Matlab command "rand". The linear fits of SVs from ten randomly generated matrixes had linear correlations between 0.92 and 0.99. Note that SVD analysis of RT in-cell NMR spectra of a labeled target protein collected over a period of 8 hours also produced no significant binding modes[1] suggesting that the analysis discriminates between true binding events and other cellular events such as random noise or dilution of the labeled protein due to cell division.

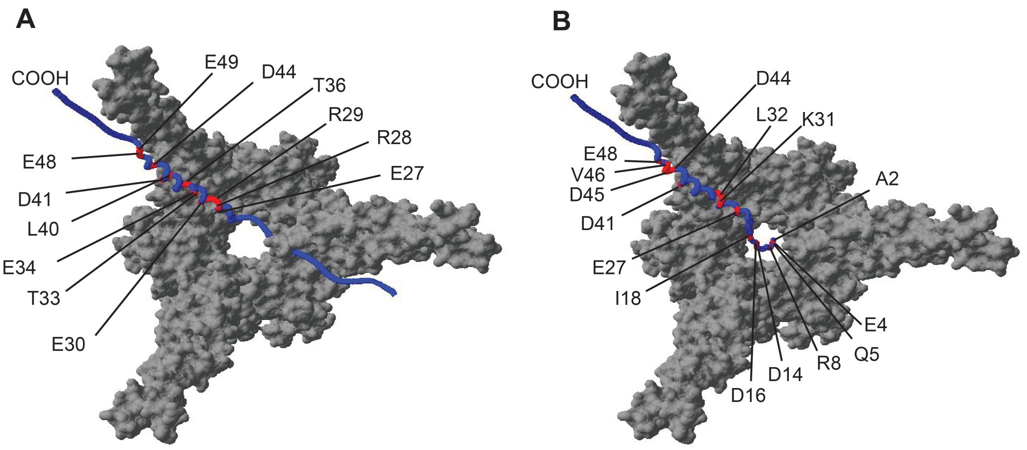

Supplementary Figure 4. Comparison between Pup-Mpa interactions as inferred from SVD analysis of in-cell NMR crosspeak intensities in metabolically inactive (A)[2] and active (B) cells.

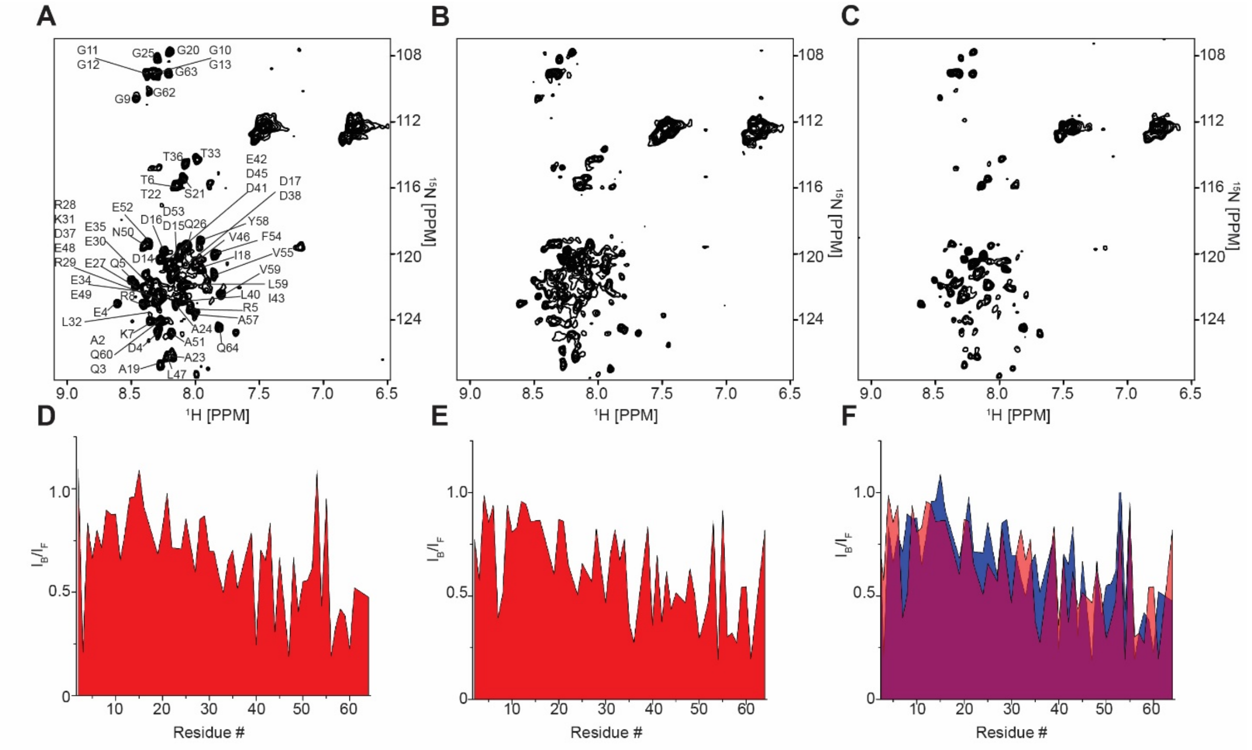

Supplementary Figure 5. *In vitro* interaction between Pup-Mpa with non-hydrolyzable ATP, AMP-PCP. ^{15}N edited HSQC spectra of [*U*- ^{15}N] labelled Pup in A) *E. coli* lysate. B) in *E. coli* lysate with 110 μM Mpa. C) in *E. coli* lysate with 110 μM Mpa and 250 μM AMP-PCP. D) Intensity ratio of Mpa-bound to free Pup. E) Intensity ratio of Mpa-bound Pup in the presence of AMP-PCP to free Pup. F) Overlay of peak intensity ratios of Mpa-bound to free Pup (blue), Mpa- bound Pup in the presence of AMP-PCP to free Pup (magenta). Non-overlapped areas of the graph are highlighted in red. All spectra are shown at the same contour level.

Supplementary References

[1] Breindel, L., DeMott, C., Burz, D. S., and Shekhtman, A. (2018) Real-Time In-Cell Nuclear Magnetic Resonance: Ribosome-Targeted Antibiotics Modulate Quinary Protein Interactions, *Biochemistry 57*, 540-546.

[2] Majumder, S., DeMott, C. M., Burz, D. S., and Shekhtman, A. (2014) Using singular value decomposition to characterize protein-protein interactions by in-cell NMR spectroscopy, *Chembiochem 15*, 929-933.
