# Supplementary material for: Active metabolism unmasks functional protein–protein interactions in real time in-cell NMR
Source: Commun Biol. 2020 May 21;3:249. doi: 10.1038/s42003-020-0976-3 (PMC7242440; doi:10.1038/s42003-020-0976-3)
Supplement: Supplementary file 2 — Description of Additional Supplementary Files [file 42003_2020_976_MOESM2_ESM.pdf]

Description of Additional Supplementary Files

File Name: **Supplementary Data 1**
Description: Matrix of in-cell NMR peak intensities of [U- 15N] Pup collected over the course of Mpa overexpression (Experiment#1).

File Name: **Supplementary Data 2**
Description: Matrix of in-cell NMR peak intensities of [U- 15N] Pup collected over the course of Mpa overexpression (Experiment#2).

File Name: **Supplementary Data 3**
Description: Data for Figure 2C, Figure 4B, and Supplementary Figure 2C.
