# Supplementary material for: Active metabolism unmasks functional protein–protein interactions in real time in-cell NMR
Source: Commun Biol. 2020 May 21;3:249. doi: 10.1038/s42003-020-0976-3 (PMC7242440; doi:10.1038/s42003-020-0976-3)
Supplement: Supplementary file 3 — Supplementary Data 1 [file 42003_2020_976_MOESM3_ESM.pdf]

Residue #	1 hrs	2hrs	3hrs	4hrs	5hrs
2	0.133853	0.112156	0.067087	0.482978	0.232065
3	0.070718	0.070924	0.079072	0.084054	0.191489
4	0.087548	0.267659	0.085797	0.25502	0.286661
5	0.139056	0.601932	0.054513	0.145821	0.333942
6	0.037107	0.135713	-0.01189	0.078828	0.089938
7	0.032782	0.089152	0.065047	0.096216	0.133987
8	0.87959	1.063731	-0.00077	0.976325	1.582165
9	0.070804	0.141098	-0.0012	0.096342	0.238593
10	0.061692	0.117985	0.020466	0.133977	0.045857
11	0.103876	0.045099	0.0716	0.1452	0.153872
12	0.120487	0.044188	0.074183	0.148945	0.130557
13	0.055519	0.134338	0.025615	0.133326	0.03893
14	0.201007	0.223081	0.05688	0.234691	0.351491
15	0.109452	0.117087	0.046837	0.291388	0.033314
16	0.124178	0.302844	0.207039	0.387146	0.200518
17	0.257414	0.130771	0.019877	0.073554	0.177561
18	0.5211	0.655133	0.06913	0.850187	0.620874
19	0.075449	0.080399	0.101398	0.121868	0.148408
20	-0.00982	0.098517	0.0559	0.079807	0.199893
21	0.116214	0.137088	0.030373	0.205179	0.086103
22	0.044736	0.23889	0.016669	0.036589	0.058804
23	0.011154	0.084109	0.039285	0.021847	0.260398
24	0.127229	0.142971	0.009509	0.147615	0.072621
25	0.138975	0.069212	0.053746	0.032853	0.073393
26	0.097817	-0.06302	0.003006	0.030659	0.041673
27	0.083198	0.130485	0.070471	0.492975	0.279335
28	0.103448	0.164742	0.044529	0.137462	0.102718
29	0.066144	0.202599	0.086884	0.179817	0.145073
30	0.078657	0.030076	-0.0258	0.215176	0.125867
31	0.070772	0.358629	0.158571	0.163856	0.139719
32	0.135684	0.274738	0.056165	0.197581	0.225631
33	0.35793	0.183154	0.11009	0.077208	0.061994
34	0.091822	0.049631	-0.01464	0.096139	0.098092
35	0.149439	0.1142	0.059974	0.199842	0.202298
36	0.202745	0.079783	0.02608	0.035475	0.399682
37	0.110625	0.175069	0.047064	0.122831	0.096017
38	0.174535	0.163884	0.070902	0.124403	0.216386
39	0.147906	0.186937	0.138532	0.210443	-0.03852
40	0.045667	0.018109	0.010031	0.103765	0.017827
41	0.11927	0.236299	0.026005	0.345785	0.279769
42	0.122153	0.145564	0.085514	0.216893	0.180548

43	0.126176	0.101711	0.097525	0.184618	-0.06249
44	0.095725	0.233845	0.114794	0.278902	0.175366
45	0.231329	0.250637	0.077812	0.208814	0.152809
46	0.257015	0.345332	-0.09539	0.439631	0.215387
47	0.101706	0.010216	0.079049	0.123786	0.143812
48	0.066144	0.351601	0.088089	0.297486	0.260178
49	0.145292	0.096337	0.035403	0.139911	0.179048
50	0.1176	0.183341	0.078352	0.1515	0.09285
51	0.041099	0.098659	0.057768	0.080221	0.080545
52	0.036484	0.116798	0.04944	0.210501	0.216481
53	0.107033	0.113207	0.045767	0.289734	0.107444
54	0.01285	0.039962	0.003653	0.170431	0.182716
55	0.088102	0.080949	0.033795	0.070059	0.137432
56	0.053338	0.036207	-0.06472	0.13228	0.073645
57	-0.00483	0.131453	-0.00201	0.159305	0.21367
58	0.177554	0.066038	0.031616	0.15219	0.070755
59	0.009288	-0.06289	0.008393	-0.08437	-0.10257
60	0.04067	0.040876	0.049025	0.054006	0.161442
61	0.102917	0.111969	0.039993	0.192067	0.077262
62	0.039236	0.025625	-0.02843	0.017576	0.12099
63	0.055566	-0.02917	0.069136	-0.02983	0.160898
64	0.001934	0.196864	-0.00539	-0.11117	-0.05315
