# Supplementary material for: Active metabolism unmasks functional protein–protein interactions in real time in-cell NMR
Source: Commun Biol. 2020 May 21;3:249. doi: 10.1038/s42003-020-0976-3 (PMC7242440; doi:10.1038/s42003-020-0976-3)
Supplement: Supplementary file 4 — Supplementary Data 2 [file 42003_2020_976_MOESM4_ESM.pdf]

Residue #	1 hrs	2hrs	3hrs	4hrs	5hrs
2	0.806959	1.652761	2.864258	1.166025	0.687141
3	0.852962	2.260913	6.198965	2.402524	1.543791
4	1.942153	1.891004	8.224058	2.359374	1.436723
5	1.378985	1.152462	4.352506	1.125533	1.450109
6	1.514989	1.058955	1.527897	2.264109	1.428732
7	0.783789	1.611699	2.861089	1.126417	0.684838
8	1.571898	1.71015	7.179772	2.516632	1.29342
9	0.480408	-0.54154	-0.38377	-1.05703	-0.40555
10	0.963901	0.706237	2.720084	0.968619	0.053434
11	0.74333	0.837489	3.589646	1.146839	-0.00933
12	0.611987	0.803798	3.564643	0.941553	0.152034
13	0.663685	0.725474	2.884252	1.022173	0.056658
14	1.148954	0.950115	3.575242	0.961736	1.18899
15	1.099036	1.05054	3.412323	0.936682	1.226736
16	0.988648	1.059115	3.519427	1.039084	0.748881
17	1.219005	1.096599	3.929118	1.067175	1.398404
18	1.174022	1.136904	3.539573	0.954747	1.219642
19	0.259273	0.58115	2.370165	0.394513	0.512363
20	1.50649	0.747393	0.081926	0.616108	-0.35032
21	1.298912	0.932675	0.552805	0.888034	0.696542
22	1.501759	1.049708	1.514555	2.244337	1.416256
23	0.383094	0.004852	0.650955	0.021917	0.124129
24	0.705229	1.3908	2.69806	2.039272	0.701425
25	0.998151	0.724068	0.005425	1.490735	-0.20588
26	1.524785	1.460994	4.714206	1.290314	1.329084
27	1.007497	1.027079	5.155896	1.728691	1.104747
28	1.530905	1.474436	6.263405	2.234206	1.119472
29	1.540138	1.470419	6.322471	2.227582	1.127878
30	1.330539	1.474161	6.457878	2.162564	1.15897
31	1.530905	1.474436	6.263405	2.234206	1.119472
32	0.748182	1.521074	2.970296	1.502042	0.790909
33	1.364103	1.258407	2.143089	1.532981	1.160758
34	1.329592	1.472539	6.39438	2.143218	1.169671
35	1.321743	1.472862	6.493011	2.174719	1.146083
36	1.385804	1.094316	1.496559	1.592807	1.073808
37	1.530905	1.474436	6.263405	2.234206	1.119472
38	1.200453	1.11682	3.660833	1.077939	1.326961
39	1.240065	1.256927	5.515376	1.792735	0.919947
40	1.324111	1.343591	1.442719	0.891032	0.572941
41	1.030854	1.011255	4.312285	1.348086	1.54468
42	1.030854	1.011255	4.312285	1.348086	1.54468

43	1.240065	1.256927	5.472812	1.85169	0.919947
44	0.121336	0.811545	2.534839	0.037192	-0.10518
45	0.91807	0.724949	4.743045	1.560345	1.287981
46	1.105091	0.98262	3.443793	0.941457	1.152625
47	0.329837	0.195165	1.03288	0.252822	0.372564
48	1.540138	1.470419	6.322471	2.227582	1.127878
49	1.330539	1.474161	6.457878	2.162564	1.15897
50	1.539477	1.518538	4.913172	1.30737	1.38235
51	0.527405	1.200518	2.829618	0.386731	0.329495
52	2.510206	0.642465	1.76868	0.116248	0.948598
53	1.136583	1.086397	3.592505	0.959375	1.157718
54	1.296822	1.150236	3.487866	1.080533	1.364273
55	1.183588	1.248797	4.960159	1.54397	1.012243
56	0.916351	1.875816	2.89999	0.641553	0.745083
57	1.732507	2.308277	1.496258	0.522683	1.036954
58	1.122581	1.202804	3.244518	0.880995	0.912975
59	0.849168	1.16872	5.012055	1.485993	0.770371
60	0.817515	2.23912	6.139213	2.379366	1.52891
61	1.381005	1.912234	3.524815	1.504614	0.796557
62	0.690839	2.917413	9.012022	2.994061	1.529347
63	0.596863	0.556506	2.185051	0.788087	0.273436
64	1.074267	1.520056	3.402162	0.579986	0.916165
