# Supplementary material for: Active metabolism unmasks functional protein–protein interactions in real time in-cell NMR
Source: Commun Biol. 2020 May 21;3:249. doi: 10.1038/s42003-020-0976-3 (PMC7242440; doi:10.1038/s42003-020-0976-3)
Supplement: Supplementary file 5 — Supplementary Data 3 [file 42003_2020_976_MOESM5_ESM.pdf]

Figure 2C:

NO FLOW

Hours	Normalized intenisity of ^{31}P-metabolites Trial1	Trial 2	Average
0	1.000	1.000	1.000
3.5	0.949	0.963	0.959
7	0.905	0.921	0.911
10.5	0.738	0.744	0.736
14	0.687	0.692	0.693
17.5	0.601	0.708	0.667
21	0.623	0.645	0.634

FLOW

Hours	Normalized intenisity of ^{31}P-metabolites Trial1	Trial 2	Average
1.5	1.000	1.000	1.000
3	1.027	1.092	1.059
4.5	1.103	1.169	1.136
6	1.122	1.185	1.153
7.5	1.154	1.227	1.190
9	1.003	1.230	1.117
10.5	1.111	1.099	1.105
12	1.120	1.080	1.100
13.5	1.127	1.270	1.198
15	1.136	1.316	1.226
16.5	1.088	1.315	1.202
18	1.113	1.356	1.235
19.5	1.049	1.305	1.177
21	1.087	1.140	1.113

Figure 4B:
Binding curve

	Binding Isotherm				
[MPA], µM	Trial 1	Trial 2	Trial 3	Average	S.D.
0.00	0.0000	0.0000	0.0000	0.0000	0.0000
6.28	0.2876	0.2430	0.2093	0.2466	0.0393
19.85	0.3887	0.3928	0.3937	0.3917	0.0027
38.80	0.6589	0.5119	0.5129	0.5612	0.0846
65.03	0.8142	0.7244	0.7163	0.7516	0.0544

81.43	0.8282	0.7487	0.7401	0.7723	0.0485
89.15	0.8536	0.7803	0.8549	0.8296	0.0427

Supplementary Figure 2C:
Size of beads

	Size of bead before ioreactor (mm)	Size of bead after Bioreactor (mm)
Trial 1	0.81	0.95
Trial 2	0.82	0.9
Trial 3	0.87	0.87
Average	0.83	0.9
S.D.	0.03	0.04
